# A Novel S-Box Design Algorithm Based on a New Compound Chaotic System

**DOI:** 10.3390/e21101004

**Published:** 2019-10-14

**Authors:** Qing Lu, Congxu Zhu, Guojun Wang

**Affiliations:** 1 Hunan Police Academy, Changsha 410138, China; hpaqlu@163.com; 2 School of Computer Science and Engineering, Central South University, Changsha 410083, China; 3 School of Computer Science, Guangzhou University, Guangzhou 510006, China

**Keywords:** chaos, S-Box, chaotic system, security, approximate entropy

## Abstract

Substitution-boxes (S-Boxes) are important non-linear components in block cryptosystem, which play an important role in the security of cryptosystems. Constructing S-Boxes with a strong cryptographic feature is an important step in designing block cipher systems. In this paper, a novel algorithm for constructing S-Boxes based on a new compound chaotic system is presented. Firstly, the new chaotic system, tent–logistic system, is proposed, which has better chaotic performance and wider chaotic range than the tent and logistic system, and can not only increase the randomness of the chaotic sequences but also expand the key space of cryptosystems. Secondly, a novel linear mapping is employed to construct the initial S-Box. Then, the permutation operation on the initial S-Box is performed by using chaotic sequence generated with the tent–logistic system, which improves the cryptographic features of the S-Box. The idea behind the proposed work is to make supplementary safe S-box. Detail tests for cryptographic strength of the proposed S-Box are performed by using different standard benchmarks. The test results and performance analysis show that our proposed S-Box has very smaller values of linear probability (LP) and differential probability (DP) and a satisfactory average value of nonlinearity compared with other S-Boxes, showing its excellent application potential in block cipher system.

## 1. Introduction

With the rapid development of network communication and big data applications, information security has become a more and more popular topic. Scholars have proposed a variety of information security technologies, including information encryption [1,2,3,4,5], watermarking [6,7], privacy protection [8,9,10], and so on. Among them, cryptography is the most basic technology in information security. In symmetric cryptographic systems, block encryption algorithms are widely used, such as in the data encryption standard (DES), advanced encryption standard (AES), and other systems. In a block cipher system, there is an important non-linear component called the substitution box (abbreviated as S-Box). S-Boxes play an important role in the security of symmetric cryptosystems. AES is considered to be an effective cryptosystem to a large extent. One of the important components of AES is its S-Box, which is based on the inversion and affine transformation of GF(2^8^) elements. Due to the popularity of AES in communication systems, S-Box has attracted more and more attention. However, the S-Box component that is used in AES is fixed. If we construct this component dynamically, the encryption strength of the cryptosystem would be greater than before.

In view of the importance of S-Box in block cipher system, the design of S-Box with strong cryptographic performance has always been the goal of cryptosystem designers. Many S-Box construction methods have been proposed [11,12,13,14,15]. In order to obtain a ciphertext block corresponding to a plaintext block, a byte conversion called the substitution byte (sub-byte) process is generated with an S-Box. In the sub-byte process, each element will be mapped using an S-Box. The S-Box is used to transform the bit input randomly. As a result, the output bit sequence has strong resistance to linear and differential attacks. Several approaches such as the analytical approach [12], algebraic techniques [14], Boolean function [16], cubic polynomial mapping methods [17], and triangle groups [18] have been applied to S-Boxes construction.

In recent years, chaotic systems have been widely applied in the design of S-Boxes because of their good cryptographic characteristics [19], such as random-like behavior [20], non-periodicity [21], and extreme sensitivity to initial conditions [22]. In [23], Lambic applied discrete chaotic map to design S-Box. In [24], Lambic proposed an efficient algorithm for obtaining random bijective S-boxes based on chaotic maps and the composition method. The advantages of Lambic’s method are the low complexity and the possibility to achieve large key space. Çavusoglu [13] designed a strong S-Box generation algorithm based on the chaotic scaled Zhongtang system. Ullah [25] constructed S-Box with the help of the chaotic system and linear fractional transformation. Belazi and El-Latif [26] proposed a simple S-Box method based on the chaotic sine map. In Ref. [27], a novel method to construct cryptographically strong bijective substitution-boxes based on a 5D hyper-chaotic system was presented. Khan et al. [28] proposed the S-Box construction method based on chaotic Boolean functions. Belazi et al. [29] proposed an efficient S-Box method based on the chaotic logistic-sine map. Wang et al. [30] constructed S-Box by using a hyper-chaotic system with infinite equilibria. Liu et al. [15] constructed S-Box based on the spatiotemporal chaotic system. 

However, these chaotic S-Box construction schemes mentioned above have not yet had a high score of linear probability (LP) and differential probability (DP), and the ability to resist linear and differential attacks were not ideal. In addition, the process of S-Box construction existing in the previous schemes is very complex and inefficient. Compared with high-dimensional continuous-time chaotic systems, low-dimensional discrete chaotic systems can generate chaotic sequences with higher efficiency. Moreover, some studies show that the complexity of discrete systems is higher than that of continuous systems [31,32,33]. However, the common low-dimensional discrete mapping chaotic systems have a narrow chaotic range and unsatisfactory chaotic characteristics. Using such chaotic systems to construct S-Boxes will reduce the key space of cryptographic systems, and the cryptographic performance is not ideal. In order to solve this problem, it is necessary to design new discrete chaotic systems with better performance.

There are two ways to implement a cryptographic system: one is software implementation, the other is hardware implementation. The hardware is quite important when trying to expand the key space of a cryptosystem. Therefore, hardware implementation is an important issue worth considering. In [34], authors provided the hardware implementation of a pseudo-random number generator (PRNG) based on three chaotic maps: the Bernoulli shift map, tent, and zigzag maps. It was found that some chaotic maps are more suitable for cryptographic applications, like the Bernoulli shift map that requires low field-programmable gate array (FPGA) resources, and provides high throughput. In [35], the authors show an application in the encryption of very high-resolution digital images based on the design of a digital chaos generator by using arbitrary precision arithmetic.

To improve the shortcomings of existing chaos based S-Box construction methods, this paper presents a novel and efficient S-Box construction method by using a new compound chaotic system. It can improve the linear probability (LP) and differential probability (DP) properties of the S-Box, and enhance the robustness of cryptosystem against linear analysis attack and differential attack. The innovations of this work are as follows:

(1) A new compound chaotic system, tent–logistic system (TLS), is proposed, which has a wider chaotic range and better chaotic performance than the old ones, so it is more suitable for cryptographic applications. 

(2) A simple and effective S-Box construction method by using a novel linear mapping and the tent–logistic chaotic system is proposed, which can improve the efficiency of S-Boxes construction. 

(3) The proposed S-Box has a higher score of LP and DP than some old S-Boxes, showing that our proposed S-Box has obvious advantages in resisting the attacks of differential cryptanalysis and linear cryptanalysis.

The rest of this paper is organized as follows. Section 2 proposes the new tent–logistic system (TLS) model. Section 3 describes the simple and effective S-Box construction method based on the tent–logistic system. Section 4 shows cryptographic performance analysis of the proposed S-Box, and makes a comparison with some recently designed S-Boxes. Section 5 completes the research paper with conclusions.

## 2. The Proposed New Chaotic System

One-dimensional (1D) discrete chaotic systems have many advantages in applications to cryptography because of their simple structures. The general mathematical model of 1D discrete mapping system can be expressed as:

(1) x(n+1)=f[x(n)],

where f[*x*] denotes functions with regard to *x*. *x*(0) is the initial state value of the system and {*x*(1), *x*(2), ...} is the output sequence of state values. For 1D discrete maps, the definition of Lyapunov exponent is as:

(2) $$\lambda =\underset{n\to \infty }{\lim}\frac{1}{n}(\sum_{i=1}^{n}\log\left|f^{\prime }[x_{i}]\right|),$$

where, f′[*x*] denotes the derivative of function f[*x*] to *x*. If *λ* > 0, then the chaotic behaviors exist in the system. In this section, we firstly reviewed two famous 1D chaotic maps: the logistic and tent chaotic maps. Then, we proposed a new discrete compound chaotic system, which has better chaotic performance and wider chaotic range than logistic and tent maps.

### 2.1. Logistic Chaotic Map

**Figure 1:**
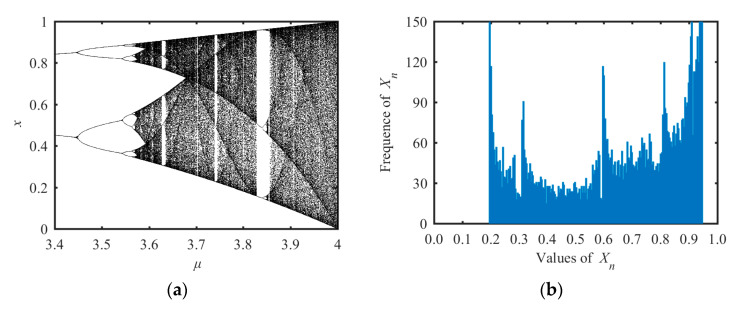
Bifurcation diagram and the state distribution of logistic system. (**a**) Bifurcation diagram and (**b**) the distribution of state values.

The logistic map is one of famous 1D chaotic maps, which has a simple mathematical structure yet complex chaotic behavior. The mathematical model of Logistic map is [36]:

(3) x(n+1)=μ×x(n)×(1−x(n)),

where *μ* is the system parameter in the range of [0, 4]. In order to determine the range of parameters corresponding to its chaotic phenomena, we calculated Lyapunov exponents under different parameters *μ* and found the chaotic rang of logistic map was *μ* ∈ [3.57, 4]. The bifurcation diagram of logistic map is shown in Figure 1a and the state distribution under *μ* = 3.78 is shown in Figure 1b.

There are three drawbacks in the logistic map. One is the chaotic range of the system is limited to *μ* ∈ [3.57, 4]. Even within this range, there are some parameters that make the logistic map to have no chaotic behaviors. Another drawback is the non-uniform distribution of state values in the range of [0, 1]. In [37], authors point out that the logistic map for *μ* = 3.9 has aperiodic behavior. Instead of using the range of 3.57 ≤ *μ* ≤ 4, one can fix the value of *μ*, however, which results in a lower key space. These drawbacks reduce the application value of the logistic map.

### 2.2. Tent Chaotic Map

**Figure 2:**
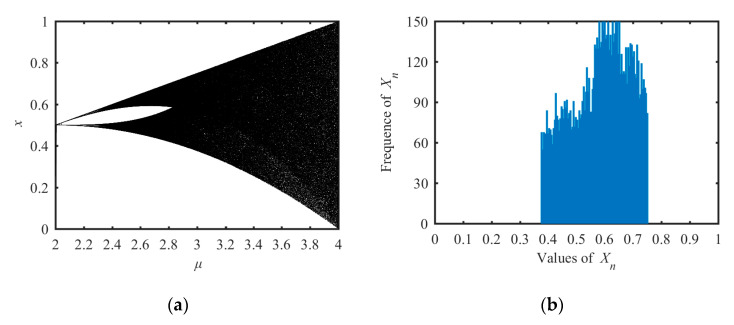
Bifurcation diagram and the state distribution of the tent system. (**a**) Bifurcation diagram and (**b**) the distribution of state values.

Tent map is another discrete 1D chaotic system, which has the tent-like shape in its bifurcation diagram. The mathematical model of the tent map is as follows [38]

(4) $$x(n+1)=\{\begin{array}{ll} f_{1}[x(n)]=\mu /2\times x(n)\; & x(n)<0.5\\ f_{2}[x(n)]=\mu /2\times (1-x(n))\; & x(n)\geq 0.5 \end{array},$$

where *μ* is the system parameter in the range of [0, 4]. By Equation (4), we could get the Lyapunov exponent of the tent map as *λ*= log(*μ*/2), so when *μ* > 2, *λ* > 0, and when *μ* = 4, *λ* = *λ_max_* = log(2) = 0.6931. Its chaotic property is shown in the bifurcation analysis in Figure 2a. Both analysis results indicate that its chaotic range was *μ* ∈ [2, 4]. The state distribution under *μ* = 3.78 is shown in Figure 2b. The tent map had the same problems as the logistic map: the small chaotic range and the no uniform distribution of the output state values.

### 2.3. The Tent–Logistic System

To solve the problems existing in logistic and tent maps, we proposed a new compound system by combining the logistic and tent maps, and called the new system the tent–logistic system (TLS). Its mathematical model is as follows:

(5) $$x(n+1)=\{\begin{array}{ll} f_{1}[x(n)]=4(9-\mu )/9\times x(n)\times (1-x(n))+2\mu /9\times x(n)\; & x(n)<0.5\\ f_{2}[x(n)]=4(9-\mu )/9\times x(n)\times (1-x(n))+2\mu /9\times (1-x(n))\; & x(n)\geq 0.5 \end{array},$$

where *μ* is the system parameter in the range of [0, 9]. When *μ* = 0, Equation (5) degenerates to the best chaotic logistic map, while *μ* = 9, Equation (5) degenerates to the best chaotic tent map. Therefore, both the best chaotic logistic and tent maps can be regarded as special cases of Equation (5).

**Proposition** **1.** *In the whole range μ ∈ [0, 9], system (5) is a map f: x_i_∈(0, 1)→x_i+1_∈(0, 1).*

**Proof.**   

(1) When *μ* = 0, Equation (5) degenerates to the chaotic logistic map f_L_: *x_i_*∈(0, 1)→*x_i_*_+1_∈(0, 1).

(2) When *μ* = 9, Equation (5) degenerates to the chaotic tent map f_T_: *x_i_*∈(0, 1)→*x_i+_*_1_∈(0, 1).

(3) When 0 < *μ* < 9 and *x*(*n*) < 0.5, f_1_′[*x*(*n*)] = (36 – 2*μ*) / 9 – (72 – 8*μ*) × *x(n) /* 9 > (36 – 2*μ*) / 9 – (72 – 8*μ*) × 0.5 / 9 = 2*μ* / 9 > 0. Hence, f_1_[*x*(*n*) < 0.5] < f_1_[0.5] = 1.

(4) When 0 < *μ* < 9 and *x*(*n*) ≥ 0.5, f_2_′[*x*(*n*)] = (36 – 6*μ*) / 9 – (72 – 8*μ*) × *x(n) /* 9 ≤ (36 – 6*μ*) / 9 – (72 – 8*μ*) × 0.5 / 9 = –2*μ* / 9 < 0. Hence, f_2_[*x*(*n*) ≥ 0.5] ≤ f_2_[0.5] = 1. f_2_[*x*(*n*) > 0.5] < f_2_[0.5] = 1. □

**Figure 3:**
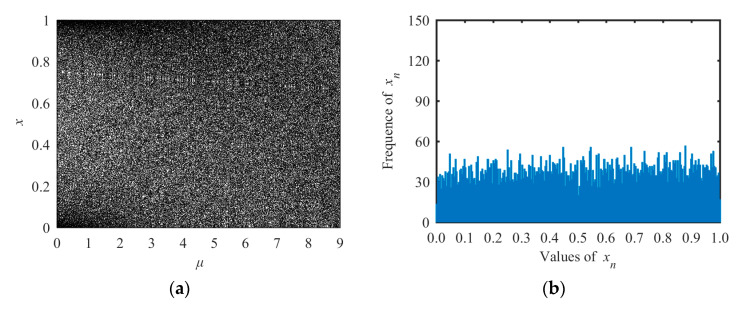
Bifurcation diagram and the state distribution of the tent–logistic system. (**a**) Bifurcation diagram and (**b**) the distribution of state values.

The bifurcation diagram and the state distribution diagram of the TLS are shown in Figure 3. From Figure 3a, one can see that the chaotic range was the whole range *μ*∈[0, 9], which was much larger than those of the logistic or tent maps. Its output sequences uniformly distributed within [0, 1] (see Figure 3b). Hence, the TLS had better chaotic performance than the logistic and tent maps.

The new tent–logistic system has two advantages compared with logistic and tent maps. First, the chaotic range of the tent–logistic system was far wider than those of the logistic and tent map. If the system parameter *μ* was used as the secret key of a cryptosystem, the key space of the cryptosystem with the new system would be much larger. Second, the output sequences of the tent–logistic system distributed evenly throughout the entire value range between 0 and 1. These advantages guarantee that the proposed tent–logistic system was more suitable for cryptographic applications.

### 2.4. Entropy Analysis of the New Chaotic System

**Figure 4:**
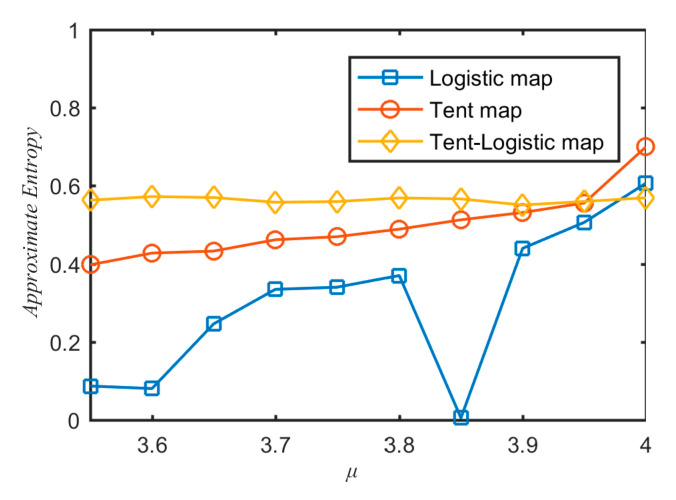
Approximate entropy values of a sequence generated by different chaotic maps.

There are many techniques to evaluate the system complexity from time sequence [39,40,41]. One of the most famous methods is approximate entropy [41]. The greater the approximate entropy, the higher the complexity of the time sequence. To measure the complexity of sequences generated by different chaotic systems, the approximate entropy values of the sequence generated by the three chaotic maps are calculated and shown in Figure 4. From Figure 4, one can see that the approximate entropy values of sequence generated by the tent–logistic map were the largest ones among the three chaotic maps in the cases of most *μ* values. It verified that the sequence generated by the tent–logistic map had larger complexity than tent and logistic maps.

### 2.5. NIST Randomness Test of PRNG with the New System

In this section, a pseudo-random number generator (PRNG) was designed by using the tent–logistic map. The specific steps of generating random number are as follows:

(1) Set the initial state value *x*_0_, the system parameter *μ*, and the positive integer *N*_0_ and *L*. 

(2) Iterate the chaotic tent–logistic map (5) *N*_0_ times to eliminate harmful effects of transient processes.

(3) Continue to iterate the chaotic tent–logistic map (5) *L* times and generate a random sequence X = [*x*_1_, *x*_2_,... *x_L_*].

(4) Through the nonlinear transformation of Equation (6), the random sequence X is transformed into the random sequence Y = [*y*_1_, *y*_2_, ..., *y_L_*].

(6) $$y_{i}=\mathrm{mod}(\mathrm{floor}(x_{i}\times 10^{14}),256),\;i\;=\;1,\;2,\ldots ,\;L,$$

where, floor(*x*) returns a maximum integer less than or equal to *x* and mod(*x*, 256) returns the remainder of *x* divided by 256. Therefore, each element in Y is an integer with the size of one byte and in the range of [0, 255], which is especially suitable for image encryption. 

(5) Transform each *y_i_* to a 8-bit binary number, then we could obtain a bit sequence B = {*b*_1_, *b*_2_, ..., *b*_8*L*_}, which is especially suitable for stream cipher application.

The random number generator test standard is the Federal Information Processing Standard issued by the National Institute of Standards and Technology (NIST). The NIST test suite includes 17 tests, which focus on a sort of different types of non-randomness that could exist in a sequence. NIST test software mainly uses two performance indicators: pass rate and *p*-value to determine the random performance of the sequence. The number of sequences to be tested is *m*, the significant level is α. If the *p*-values of *N* sequences are greater than α, then the pass rate is *N*/*m*. The default value of α is 0.01. To test the random performance of the bit sequences generated by our PRNG, we set the parameters as: *x*_0_ = 0.66, *μ* = 4.5, *L* = 12.5 × 10^6^, and *N*_0_ = 500. Then, a bit sequence was generated, which had the length of 100 × 10^6^ bits. The bit sequence was divided into 100 sub-sequences of equal length, each of which was 10^6^ bits in length. By this way, 100 sequences of 10^6^ bits were produced. The results from all statistical tests are given in Table 1. The min *p*-value in Table 1 was 0.045675, which was larger than 0.01. The minimum pass rate for each statistical test was 98 for 100 binary sequences. Therefore, the sequence generated with the new tent–logistic system and the generation algorithm could be considered to have high randomness. It is worth noting that the smaller number of sequences was used for random excursions tests in the Table 1. It was due to the fact that random excursions and random excursions variant tests were not applicable to binary sequences with insufficient number of cycles. Therefore, only samples with the number of cycles exceeding 500 were evaluated for these tests. In our test, there were 57 samples with the number of cycles exceeding 500.

## 3. Proposed New S-Box Design

### 3.1. Introduction of S-Boxes

**Figure 5:**
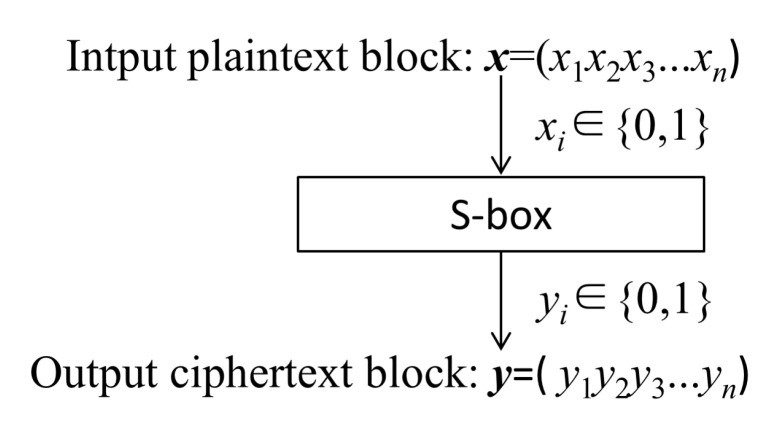
The function and basic principle of an *n* × *n* S-Box.

An S-Box is the only non-linear component in a block cipher system. It plays an important role in symmetric block cipher cryptosystems. An S-Box is like a black box. It transforms any input plaintext block into a ciphertext block, which can confuse the relationship between ciphertext and plaintext. A general *m* × *n* S-Box is a map: {0, 1}*^m^*→{0, 1}*^n^*. When *n* = *m*, it means that data are neither compressed nor expanded during encryption transformation. In this case of *n* = *m*, the S-Box can realize completely reversible transformation. Most S-Boxes commonly used in cryptography are *m* = *n*. The function and basic principle of an *n* × *n* S-Box can be shown in Figure 5. 

An *n* × *n* S-Box is a number set of {0, 1, 2, ..., 2*^n^* − 1}, which is represented by a 2*^n/2^* × 2*^n/2^* matrix Sb. S boxes that are 8 × 8 are the most commonly used type of S-Box, especially widely used in digital image encryption system [42]. In this paper, we focused on the design algorithm of an 8 × 8 S-Box. An 8 × 8 S-Box is a number set of {0, 1, 2, ..., 255}, which is represented by a 16 × 16 matrix Sb = {Sb(*i*, *j*)|*i* = 1, 2,..., 16; *j* = 1, 2, ..., 16} shown in Table 2. For 8 × 8 S-Boxes, there are a total of ($2^{8}!$) different forms of variation. Among ($2^{8}!$) different forms of variation, the simplest 8 × 8 S-Box arranges elements in an orderly manner from small to large values. As the result, elements in the simplest 8 × 8 S-Box has the form: Sb(*i*, *j*) = (*i*-1) × 16 + *j*-1.

The process of converting plaintext byte *x* into ciphertext byte *y* through an S-Box with matrix Sb can be expressed by the function S[*x*] as:

(7a) $$\left\{ \begin{array}{l} i=\mathrm{floor}(x/16)+1,j=\mathrm{mod}(x,16)+1\\ y=S[x]=\mathrm{Sb}(i,j) \end{array}\right.,$$

where, floor(*a*) rounds *a* to the nearest integer less than or equal to *a*. mod(*a*, *m*) returns the remainder after division of *a* by *m*, where *a* is the dividend and *m* is the divisor. Equation (7a) is a process in which each pixel value in a plain image is substituted with an element value in the S-Box. For example, if *x* = 55, then *i* = floor(55/16) + 1 = 3 + 1 = 4, *j* = mod (55, 16) + 1 = 7 + 1 = 8. Consequently, *y* = S[*x*] = Sb(*i*, *j*) = Sb(4, 8). For the simplest 8 × 8 S-Box, we could obtain the following results easily as: S[0] = Sb(1, 1) = 0, S[1] = Sb(1, 2) = 1, ..., S[255] = Sb(16, 16) = 255. Namely, *y* = S[*x*] = *x*. It is obvious that the simplest 8 × 8 S-Box can not alter any input plaintext value, so the simplest 8 × 8 S-Box can not be used in the encryption system.

Corresponding to the transformation *y* = S[*x*] in the encryption procedure, we defined the inverse transformation *x* = $S^{-1}[y]$ in the decryption procedure. The steps of calculating $S^{-1}[y]$ are as follows:

(7b) $$\left\{ \begin{array}{l} \mathrm{Find}\;i\;\mathrm{and}\;j\;\mathrm{in}\;\mathrm{Sb}\;\mathrm{such}\;\mathrm{that}\;\mathrm{Sb}(i,j)=y\\ x=S^{-1}[y]=(i-1)\times 16+j-1 \end{array}\right..$$

### 3.2. The Proposed Algorithm for Generating S-Box 

Many researchers have done extensive research on the design methods of S-Boxes with different cryptographic strength. However, most of these methods are complex and inefficient, so the time cost of generating S-Boxes is large. Here, we proposed a very simple and efficient design methods to construct strong S-Boxes based on the new chaotic map and a nonlinear mapping. The new method takes advantage of the excellent chaotic characteristics of the tent–logistic map. The detailed steps of generating new S-Boxes are given below.

Step 1: Set a integer parameter *A* such that *A* > 0 and *A* ≠ *k* × 257, *k* = 1, 2, 3,....

Step 2: Let T ← [0, 1, 2, ..., 255], then we obtained an array T, which contained 256 distinct integers in the range of [0, 255].

Step 3: Based on T and *A* to obtain a new array R by the following linear mapping:

*R*(*i*) = mod((*A*×(*T*(*i*)+1)), 257), *i* = 1, 2, ..., 256 (8)

where *T*(*i*)∈{0, 1, ...,255}, *A* is a positive integer satisfying *A* ≠ *k* × 257, and *k* is a positive integer. (*A*/257) is not an integer, and (*T*(*i*)+1)/257 is also not an integer. As a result, (*A*×(*T*(*i*)+1)) cannot be divided exactly by 257. Namely, mod((*A*×(*T*(*i*)+1)), 257) ≠ 0. Therefore, Equation (8) is a map: *T*(*i*)∈{0,1,2,...,255} → *R*(*i*)∈{1, 2, ..., 256}. 

Step 4: Let *R*(*i*) ← *R*(*i*) – 1, then *R*(*i*)∈{0, 1, ..., 255}, *i* = 1, 2, ..., 256. We obtained a 1D array **R** = {*R*(*i*)}. 

Step 5: Transform the 1D array R into a 2D matrix Rb, and then Rb could be considered as the initial S-Box.

Step 6: Set the parameters *μ*, initial state value *x*_0_ of the tent–logistic map, and an integer *L* that was far larger than 256. Then iterate the tent–logistic map *L* times to generate a chaotic sequence of length *L*. In order to improve the sensitivity of output chaotic sequence to its initial state value, we discarded the first (*L*-256) elements of the original chaotic sequence, and then we could obtain a new chaotic sequence of length 256, which is represented by X. 

Step 7: Sort the chaotic sequence **X**, then we could get a position index array J = {J(1), J(2), ..., J(256)}, J(*i*)∈{1, 2, ..., 256}. Due to the non-periodicity and ergodicity of chaotic sequences, it will inevitably lead to that J(*i*)≠J(*j*) as long as *i*≠*j*.

Step 8: Calculate the 1D array S1 as follows:

S1(*i*) = T(J(*i*)), *i* = 1, 2,..., 256. (9)

Step 9: Transform the 1D array S1 into a 2D matrix Sb, and this was the proposed S-Box. 

By the proposed method, the length of chaotic sequences to be used in constructing a 16 × 16 sized S-Box matrix was 256. The purpose of taking *L* far larger than 256 is to execute (*L*-256) times pre-iterations, which could enhance the sensitivity of S-Box to the initial value *x*_0_ of the chaotic system. In the process of concrete realization, the proposed new S-Box was generated by the above S-Box generation algorithm with parameters were set as {*x*_0_ = 0.66, *μ* = 4.5, *A* = 56, *L* = 65536}, which is shown in Table 3. The number in the first row of Table 3 represents the column number of the S-Box matrix, while the number in the first column of Table 3 represents the row number of the S-Box matrix.

## 4. Performance Tests

In this section, we tested the cryptographic strength of our proposed new S-Box given in Table 3 with widely used standard S-Box performance evaluation criteria.

### 4.1. Bijectiveness

A function S: *x*∈N → *y*∈N is bijective if and only if it is one-to-one map. From Equation (7a) and Table 3, it is obvious that one can obtain a distinct value *y*∈N corresponding to a certain *x*∈N. Conversely, from a certain *y*∈N, one can find a distinct value in the matrix SB that equal to *y* and obtain a distinct value pair (*i*, *j*). By the inverse transformation S^−1^[*y*] defined by Equation (7b), one can obtain a distinct value *x*. Therefore, function S corresponding to S-Box of Table 3 is bijective.

### 4.2. Strict Avalanche Criterion (SAC)

The strict avalanche criterion (SAC) [11,12] is a crucial feature for any cryptographic S-Box. SAC requires that if a single *j*-th bit in the input value *x* is changed, the probability of causing the change of the *i*-th bit in the output cipher text value *y* should be 0.5. Namely, the probability *p*(*i*, *j*) should be 0.5 for all *i* = 1, 2, ..., *n*, and *j* = 1. 2, ..., *n*. An S-Box having *p*(*i*, *j*) values of SAC closer to 0.5 has satisfactory uncertainty. Dependency matrix providing the SAC values of an S-Box. Table 4 listed the dependency matrix of the proposed S-Box for strict avalanche criterion (SAC). The values corresponding to the positions of *i*-th row and *j*-th column in the table are *p*(*i*, *j*) values. It is evident from Table 4 that *p*(*i*, *j*) values of the S-Box was very close to 0.5 (an average value of *p*(*i*, *j*) was 0.505), showing that the proposed S-Box satisfied the SAC criterion.

### 4.3. Nonlinearity

The nonlinear mapping of an S-Box can also be expressed as:

(10) $$y=y_{1}y_{2}\ldots y_{n}=S[x]=S_{1}[x]S_{2}[x]\ldots S_{n}[x],$$

where, $y_{i}=S_{i}[x]\in \{0,1\}$ and $S_{i}[x]$ is an *n*-bit Boolean function, *i* = 1, 2, ..., *n*. In order to effectively resist linear cryptanalysis attack, an S-Box must have high nonlinear relationship between its input and output values. The nonlinearity of an *n*-bit Boolean function $S_{i}[x]$ is used to measure the nonlinear strength of an *n* × *n* S-Box, which can be calculated by:

(11) $$NL_{i}=\frac{1}{2}(2^{n}-\underset{x\in {\left\{0,1\right\}}^{n}}{\mathrm{Max}}\left|\mathrm{WS}\_S_{i}[x]\right|),$$

where, WS_S*_i_*[*x*] is the Walsh spectrum of function S*_i_*[*x*], and it is calculated as:

(12) $$\mathrm{WS}\_S_{i}[x]={\sum_{z\in {\left\{0,1\right\}}^{n}}\left(-1\right)}^{S_{i}[x]⊕x\cdot z},$$

where, x·z denotes the dot product of *x* and *z*, which is calculated as:

(13) $$x\cdot z=(x_{1}\times z_{1})⊕(x_{2}\times z_{2})⊕\cdots ⊕(x_{n}\times z_{n}),$$

where ⊕ denotes the modulo 2 addition. *NL_i_* is the nonlinearity value of the *i*-th constituent Boolean functions in an S-Box. The larger the nonlinearity, the better the performance of an S-Box against linear cryptanalysis attack. The nonlinearity values of all eight constituent Boolean functions in the proposed S-Box are listed in Table 5. The minimum of nonlinearity was 104, the maximum of nonlinearity was 110, and the average value of nonlinearity was 106.3. Table 5 also lists the nonlinearity values of the initial S-Box, the nonlinearity values of which were much less than those of the final S-Box. The results show that the nonlinearity of the final S-Box was greatly improved by introducing chaotic sequence to scramble the initial S-Box.

### 4.4. Bit Independence Criterion (BIC)

According to the criterion of BIC [11,12], when the *k*-th bit of the input data block changes (flips), the *i*-th bit and *j*-th bit of the output data block changes independently (or without any dependence on each other). Then it means that the response of the output bit values of the S-Box to the change of an input bit is independent. To measure this feature of an S-Box, the bit independence criterion for strict avalanche criterion (BIC–SAC) was introduced. To determine the BIC–SAC results, we could calculate the sum of ${(S}_{i}[x]⊕S_{j}[z]-S_{i}[x]⊕S_{j}[x])$ for all input *x*∈{0, 1, ..., 255}, where *z* and *x* were only one bit different every time. If the average BIC–SAC values for all input *x*∈{0, 1, ..., 255} were close to 0.5, and then the S-Box met BIC–SAC very well. For our proposed S-Box, the BIC–SAC results are listed in Table 6, which had the average value 0.49937. The results show that our proposed S-Box met BIC–SAC very well. 

Another indicator of bit independence criterion was the BIC results for nonlinearity. To determine the BIC results for nonlinearity, we could calculate the nonlinearity values for each output bit value of ${(y}_{i}⊕y_{j})$ for all input *x*∈{0, 1, ..., 255}, where *i* = 1, 2, ..., *n* and *j* = 1, 2, ..., *n*. For our proposed S-Box, the BIC results for nonlinearity are listed in Table 7, which had the average value 103.8. The experimental results show that our proposed S-Box met BIC for nonlinearity very well. 

It is crystal clear from Table 6 and Table 7 that average SAC and nonlinearity values for BIC were 0.499 and 103.8, respectively. According to Ref. [12], if an S-Box exhibits SAC and nonlinearity, it fulfills BIC. The obtained scores of 0.499 and 103.8 for our proposed S-Box clearly manifested an exceedingly strong nonlinearity interrelation among the output bits. These test results fully validated BIC of our proposed S-Box.

### 4.5. Linear Probability

A secure cryptosystem should have strong confusion and diffusion effects. Strong S-Boxes help cryptosystems to achieve strong confusion and diffusion effects through nonlinear mapping between input and output data. The lower linear probability (LP) of an S-Box, the higher the nonlinear mapping feature and the stronger the performance resistance against the linear cryptanalysis. Therefore, linear probability (LP) was used to measure the resistance of an S-Box to linear cryptanalysis, which was calculated by:

(14) $$LP=\underset{\alpha_{x},\beta_{x}\neq 0}{\mathrm{Max}}\left|\frac{\#\{x\in N|x\cdot \alpha_{x}=S(x)\cdot \beta_{x}\}}{2^{n}}-\frac{1}{2}\right|,$$

where, N = {0, 1, ... , 255}, $\alpha_{x}$ and $\beta_{x}$ are the corresponding input and output masks ($\alpha_{x}$∈N, $\beta_{x}$∈N), “.” denotes the dot product operation mentioned above, and #{*x*∈N|X} denotes the number of *x* satisfying the condition X. The maximal value of LP of our proposed S-Box was only 0.125, and thus provides good resistance against linear cryptanalysis.

### 4.6. Differential Probability

Differential cryptanalysis [43] is another effective method to decipher ciphertext. This method is to find the plaintext pairs and corresponding ciphertext pairs having the same differentials. By these plaintext pairs and corresponding ciphertext pairs, the attackers can gain some part of the key. In order to measure the performance of S-Box against differential cryptanalysis, the differential probability (DP) is introduced, which is calculated by:

(15) $$DP=\underset{\Delta x\neq 0,\Delta y}{\mathrm{Max}}(\frac{\#\{x\in N|S(x)⊕S(x⊕\Delta x)=\Delta y\}}{2^{n}}),$$

where, $\Delta x=x⊕x^{’}$ and $\Delta y=y⊕y^{’}$ are differentials corresponding to input pair (*x*, *x*’) and output pair (*y*, *y*’), respectively. An S-Box with smaller differential probability (DP) has a stronger ability to resist differential cryptanalysis. The maximal value of DP of our proposed S-Box was only 0.039. This small value indicates that the proposed S-Box had strong resistance to differential cryptanalysis attacks.

### 4.7. Performance Comparison

In order to compare the cryptographic performance of S-Boxes proposed in this paper with some recently proposed S-Boxes, the performance index values of these S-Boxes are listed in Table 8. From Table 8, it can be seen that our S-Box had the smaller values of LP and DP than most of the other S-Boxes in Table 8. The results show that our S-Box had obvious advantages in resisting the attacks of differential cryptanalysis and linear cryptanalysis. Our S-Box had an average value of nonlinearity greater than most of the other S-Boxes in Table 8. The results also indicate that the SAC value (0.505) of our proposed S-Box was very near to the ideal value of SAC (0.5). The BIC value of the proposed S-Box was also quite good ensuing gratification of the BIC test. It is worth noting that the initial S-Box obtained by this algorithm has poor nonlinearity (see the penultimate row of Table 8). By introducing the chaotic sequence to disturb the initial S-Box, the final S-Box obviously enhanced the nonlinearity. In our opinion, it is very important for randomly generated S-boxes to obtain similar quality as good S-boxes. In addition to these quality evaluation indicators, the novelty, time, and space overhead of the algorithm are also very important evaluation criteria.

## 5. Conclusions

The application of a new chaotic system and novel linear mapping for constructing S-Boxes was presented in this paper. The innovations of this work were as follows:

- A new compound chaotic system, the tent–logistic system (TLS), was proposed, which had a wider chaotic range and better chaotic performance than the old ones. The TLS could not only increase the randomness of the constructed S-Box but also expanded the key space of cryptosystems.
- A simple and effective S-Box construction method was proposed. The novel linear mapping was employed to construct the initial S-Box and the TLS was used to scramble the initial S-Box. The efficiency of constructing S-Boxes was higher and the cryptographic features of the S-Box were better.
- The proposed S-Box had a higher score of LP and DP than some old S-Boxes, showing that our proposed S-Box had obvious advantages in resisting the attacks of differential cryptanalysis and linear cryptanalysis.

Detail tests for cryptographic strength of the proposed S-Box were performed by using different standard benchmarks. The test results and performance analysis show that our proposed S-Box had very smaller values of LP and DP and a satisfactory average value of nonlinearity compared with other S-Boxes. It means that our proposed S-Box provided good resistance against linear cryptanalysis and differential cryptanalysis and had potential in the block cipher system.

In the future research, we think that it is possible to optimize this S-Box based on the tent–logistic map applying metaheuristics, similar to the optimization performed for continuous chaotic systems as shown in [46]. In addition, we could apply this S-Box in designing image encryption schemes.

## Figures and Tables

**Table 1:** Results of applying National Institute of Standards and Technology (NIST) test to our pseudo-random number generator (PRNG).

NIST Statistical Test	*p*-Value	Pass Rate	Results
Frequency (monobit)	0.911413	99/100	pass
Block Frequency (m = 128)	0.897763	99/100	pass
Cumulative Sums (Forward)	0.637119	100/100	pass
Cumulative Sums (Reverse)	0.779188	100/100	pass
Runs	0.202268	100/100	pass
Longest Run of Ones	0.897763	98/100	pass
Rank	0.401199	100/100	pass
FFT	0.574903	98/100	pass
Non-Overlapping Templates (m = 9, B = 000000001)	0.045675	99/100	pass
Overlapping Templates (m = 9)	0.834308	99/100	pass
Universal	0.236810	98/100	pass
Approximate Entropy (m = 10)	0.574903	99/100	pass
Random-Excursions	0.554420	57/57	pass
Random-Excursions Variant	0.474986	56/57	pass
Serial Test 1 (m = 16)	0.096578	100/100	pass
Serial Test 2 (m = 16)	0.935716	99/100	pass
Linear complexity (M = 500)	0.090936	100/100	pass

**Table 2:** The matrix Sb of an 8 × 8 S-Box.

*i*/*j*	1	2	3	...	15	16
**1**	Sb(1,1)	Sb(1,2)	Sb(1,3)	⋯	Sb(1,15)	Sb(1,16)
**2**	Sb(2,1)	Sb(2,2)	Sb(2,3)	⋯	Sb(2,15)	Sb(2,16)
**3**	Sb(3,1)	Sb(3,2)	Sb(3,3)	⋯	Sb(3,15)	Sb(3,16)
**⋮**	⋮	⋮	⋮	Sb(*i*, *j*)	⋮	⋮
**15**	Sb(15,1)	Sb(15,2)	Sb(15,3)	⋯	Sb(15,15)	Sb(15,16)
**16**	Sb(16,1)	Sb(16,2)	Sb(16,3)	⋯	Sb(16,15)	Sb(16,16)

**Table 3:** The proposed new S-Box.

*i*/*j*	1	2	3	4	5	6	7	8	9	10	11	12	13	14	15	16
**1**	114	75	39	161	61	14	225	150	180	126	232	155	171	129	143	26
**2**	186	76	234	247	53	185	187	227	106	192	99	31	94	215	219	20
**3**	110	105	112	60	52	90	188	221	8	48	208	107	201	24	212	19
**4**	49	191	91	138	97	238	140	220	122	63	139	146	167	137	28	88
**5**	135	4	222	18	36	168	181	32	9	117	83	148	190	127	102	236
**6**	205	82	121	199	252	147	67	133	204	111	98	210	173	243	1	184
**7**	174	230	59	30	176	21	160	62	202	145	195	209	119	96	45	141
**8**	245	44	78	29	43	177	12	194	156	38	151	50	213	244	22	142
**9**	170	226	101	72	152	115	217	2	163	109	239	37	104	196	3	189
**10**	198	218	57	124	27	134	175	74	87	108	89	224	125	237	65	118
**11**	197	5	158	66	42	157	229	255	211	207	55	203	169	123	56	149
**12**	242	254	200	100	95	69	46	23	40	251	7	6	103	216	178	79
**13**	240	253	131	15	183	113	246	93	71	153	249	77	248	10	172	250
**14**	35	41	132	25	33	47	223	86	81	154	136	233	13	68	64	54
**15**	166	120	84	17	193	214	0	85	73	92	70	164	182	16	206	130
**16**	144	228	11	179	80	159	116	128	235	51	241	165	34	231	162	58

**Table 4:** Dependency matrix of the proposed S-Box for the strict avalanche criterion (SAC).

*i*/*j*	1	2	3	4	5	6	7	8
**1**	0.5156	0.5000	0.4688	0.5156	0.5313	0.5156	0.5469	0.5156
**2**	0.5313	0.5313	0.5000	0.4688	0.5156	0.4375	0.4375	0.4219
**3**	0.6250	0.5313	0.5156	0.5313	0.5000	0.4688	0.5469	0.5000
**4**	0.5625	0.4688	0.6094	0.4375	0.3906	0.5156	0.4531	0.5625
**5**	0.5156	0.5000	0.5313	0.5000	0.5313	0.5000	0.5156	0.5000
**6**	0.5156	0.4844	0.5156	0.5469	0.5156	0.4688	0.4531	0.4688
**7**	0.4844	0.5000	0.5156	0.5156	0.5313	0.4688	0.4531	0.4844
**8**	0.4844	0.4844	0.5313	0.5000	0.5469	0.5156	0.5469	0.5313

**Table 5:** Nonlinearities of constituent Boolean functions of the proposed S-Box.

S-Box/S*_i_*	S_1_	S_2_	S_3_	S_4_	S_5_	S_6_	S_7_	S_8_	Average
Initial S-Box	54	54	54	54	54	54	54	54	54
Final S-Box	108	106	104	104	104	106	108	110	106.3

**Table 6:** Bit independence criterion for SAC.

Boolean Function	S_1_	S_2_	S_3_	S_4_	S_5_	S_6_	S_7_	S_8_
**S_1_**	-	0.4785	0.4707	0.4941	0.5098	0.4902	0.5137	0.5117
**S_2_**	0.4785	-	0.5215	0.4902	0.5254	0.5039	0.4902	0.5098
**S_3_**	0.4707	0.5215	-	0.5215	0.4980	0.4961	0.4980	0.5020
**S_4_**	0.4941	0.4902	0.5215	-	0.4727	0.4941	0.5117	0.4961
**S_5_**	0.5098	0.5254	0.4980	0.4727	-	0.4766	0.5156	0.5098
**S_6_**	0.4902	0.5039	0.4961	0.4941	0.4766	-	0.4805	0.5059
**S_7_**	0.5137	0.4902	0.4980	0.5117	0.5156	0.4805	-	0.4941
**S_8_**	0.5117	0.5098	0.5020	0.4961	0.5098	0.5059	0.4941	-

**Table 7:** Bit independence criterion for nonlinearity.

Output bit pair Function	y_1_	y_2_	y_3_	y_4_	y_5_	y_6_	y_7_	y_8_
**y_1_**	-	102	106	106	104	106	100	102
**y_2_**	102	-	104	102	98	102	108	108
**y_3_**	106	104	-	106	104	106	104	106
**y_4_**	106	102	106	-	104	100	106	102
**y_5_**	104	98	104	104	-	100	102	106
**y_6_**	106	102	106	100	100	-	102	104
**y_7_**	100	108	104	106	102	102	-	106
**y_8_**	102	108	106	102	106	104	106	-

**Table 8:** Performance comparison of different S-Boxes.

S-Box Method	SAC	Nonlinearity Min. Max. Average			BIC–SAC	BIC-NL	LP	DP
Ref. [1]	0.495	104	110	106.5	0.498	103.8	0.141	0.039
Ref. [15]	0.498	102	108	104.5	0.508	104.6	0.125	0.047
Ref. [17]	0.507	104	108	106.8	0.507	103.9	0.140	0.054
Ref. [23]	0.503	106	108	106.8	0.502	103.8	0.133	0.039
Ref. [24]	0.501	108	112	109.3	0.506	108.2	0.094	0.031
Ref. [29]	0.496	102	108	105.3	0.499	103.8	0.156	0.039
Ref. [30]	0.520	104	110	106.3	0.501	104.2	0.133	0.039
Ref. [44]	0.502	102	108	103.5	0.501	103.0	0.133	0.039
AES [45]	0.504	112	112	112	0.504	112	0. 062	0.016
Initial S-Box	0.438	54	54	54	0.501	77.1	0.289	1.000
Final S-Box	0.505	104	110	106.3	0.499	103.8	0.125	0.039
